# Anodal transcranial direct current stimulation over the left temporal pole restores normal visual evoked potential habituation in interictal migraineurs

**DOI:** 10.1186/s10194-017-0778-2

**Published:** 2017-07-19

**Authors:** Francesca Cortese, Francesco Pierelli, Ilaria Bove, Cherubino Di Lorenzo, Maurizio Evangelista, Armando Perrotta, Mariano Serrao, Vincenzo Parisi, Gianluca Coppola

**Affiliations:** 1 Department of Medico-Surgical Sciences and Biotechnologies, Sapienza University of Rome Polo Pontino, Corso della Repubblica, 79 – 04100, Latina, Italy; 2 INM Neuromed IRCCS, Pozzilli (IS), Italy; 3 Don Carlo Gnocchi, Onlus Foundation, Milan, Italy; 4 Università Cattolica del Sacro Cuore/CIC, Istituto di Anestesiologia, Rianimazione e Terapia del Dolore, Rome, Italy; 5 G. B. Bietti Foundation IRCCS, Research Unit of Neurophysiology of Vision and Neuro-Ophthalmology, Rome, Italy

**Keywords:** Ventral stream, Visual system, Somatosensory system, Synaptic plasticity, Neurostimulation

## Abstract

**Background:**

Neuroimaging data has implicated the temporal pole (TP) in migraine pathophysiology; the density and functional activity of the TP were reported to fluctuate in accordance with the migraine cycle. Yet, the exact link between TP morpho-functional abnormalities and migraine is unknown. Here, we examined whether non-invasive anodal transcranial direct current stimulation (tDCS) ameliorates abnormal interictal multimodal sensory processing in patients with migraine.

**Methods:**

We examined the habituation of visual evoked potentials and median nerve somatosensory evoked potentials (SSEP) before and immediately after 20-min anodal tDCS (2 mA) or sham stimulation delivered over the left TP in interictal migraineurs.

**Results:**

Prior to tDCS, interictal migraineurs did not exhibit habituation in response to repetitive visual or somatosensory stimulation. After anodal tDCS but not sham stimulation, migraineurs exhibited normal habituation responses to visual stimulation; however, tDCS had no effect on SSEP habituation in migraineurs.

**Conclusion:**

Our study shows for the first time that enhancing excitability of the TP with anodal tDCS normalizes abnormal interictal visual information processing in migraineurs. This finding has implications for the role of the TP in migraine, and specifically highlights the ventral stream of the visual pathway as a pathophysiological neural substrate for abnormal visual processing in migraine.

## Background

Migraine is a neurological disorder that is characterized by recurrent clinical attacks separated by variable-length headache-free intervals. Although the pathogenesis of migraine is far from completely understood, clinical neurophysiology and neuroimaging studies in recent decades have disclosed subtle functional and morphological abnormalities that manifest during the interictal phase and distinguish migraineurs from normal healthy subjects [1–3]. Among the various subcortical and cortical areas implicated in migraine pathophysiology, emerging evidence highlights the temporal pole (TP) as a key neural substrate. In humans, the TP serves as a multimodal neural hub that receives and integrates various sensory modalities including olfactory, auditory, taste, and visual inputs. Moreover, the TP participates in the ventral visual stream (VVS) for visual information processing [4–6]. During an olfactory task, interictal migraineurs exhibited significantly higher brain glucose metabolism in the left TP compared to control subjects [7]. Moreover, BOLD signal in the TP in response to noxious stimulation was reduced in interictal patients compared to patients who were actively experiencing a migraine [8, 9]. In resting-state MRI studies comparing interictal migraineurs to healthy control subjects, decreased grey matter density was observed in the left TP [10] and the left TP exhibited decreased connectivity with components of the default-mode network [11]. Finally, the TP was implicated as an important area for differentiating patients with migraine from healthy control subjects in a cross-sectional brain MRI investigation [12]. Taken together, these findings suggest that the TP is both intricately related to the pathophysiology of migraine and sensitive to the cyclical recurrence of migraine attacks.

Transcranial direct current stimulation (tDCS) is a non-invasive technique for neuromodulation in humans that affects cortical excitability in a polarity-specific manner [13, 14]. Anodal polarization increases the excitability of cortical areas below electrodes, whereas cathodal polarization typically decreases cortical excitability [15]. A number of tDCS studies in different pain disorders [16, 17] have demonstrated that tDCS is well-tolerated by patients [18]. Anodal tDCS proved effective over either the motor cortex or the dorsolateral prefrontal cortex when used as prophylactic strategy both in episodic [19] and chronic [20, 21] migraine. Moreover, some studies reported that, in addition to the therapeutic effects, tDCS over the visual cortex also normalized interictal cortical hyperresponsivity in episodic migraine [22].

Nonetheless, to our knowledge, no study to date has targeted the TP for anodal tDCS in migraine, to enhance interictal temporal lobe activity and thereby interfere with an aspect of migraine pathophysiology. Thus, we examined whether anodal stimulation of the TP could restore normal function of the TP and thus physiological information processing in migraine. Moreover, given that the TP processes all kinds of sensorial information except for somatosensory information, we examined the habituation responses of evoked potentials to somatosensory stimuli (as a negative control) as well as visual stimuli.

## Methods

### Participants

Forty patients with migraine without aura (diagnosed in accordance with the International Classification of Headache Disorders III beta edition) were recruited from our headache clinic (Table 1). Of these, 4 patients were excluded because they did not meet the primary inclusion criteria (see below). Subjects were included if they were between 18 and 65 years of age and had at least a 1-year clinical history of migraine with 2–8 attacks per month. The use of preventive anti-migraine medication was not permitted during the 3 months preceding the study. The primary inclusion criterion was being attack-free for at least 3 days before and after each recording sessions, and was verified by headache diary and telephone or e-mail interview. Subjects were excluded from the study if they were regularly taking medication (e.g., antibiotics, corticosteroids, antidepressants, benzodiazepines, or prophylactic migraine medication) except for contraceptive pills; if they did not have a best-corrected visual acuity of >8/10; and if they had a history of other neurological disease, systemic hypertension, diabetes or other metabolic disease, autoimmune disease, or any other type of primary or secondary headache. Female participants were always recorded mid-menstrual cycle. All participants received a complete description of the study and provided written informed consent. The study was approved by a local ethical review board and was conducted in accordance with the Helsinki Declaration.

**Table 1:** Descriptive statistics of clinical and demographic characteristics of migraine patients between attacks in the sham and real group

	Real (*n* = 18)	Sham (*n* = 18)	*p* value
Women (n)	13	11	0.495
Age (years)	28.6 ± 7.6	26.9 ± 4.9	0.430
Duration of migraine history (years)	15.6 ± 8.3	12.4 ± 7.0	0.220
Attack frequency/month (n)	5.0 ± 3.2	3.9 ± 2.1	0.231
Attack duration (hours)	17.1 ± 17.4	18.1 ± 14.8	0.854
Visual analogue scale (n)	7.0 ± 0.7	6.6 ± 1.2	0.230
Days from the last migraine attack (n)	8.5 ± 8.5	11.7 ± 13.0	0.388
Family history of migraine (%)	51.4	48.6	0.210
Acute medication intake/month (n)	2.0 ± 1.9	2.0 ± 1.8	0.996

Data are expressed as means ± SD

### Experimental procedure

The 36 enrolled patients were equally randomized to receive anodal tDCS (*N* = 18) or sham tDCS (*N* = 18). Randomization was conducted using a secure web-based database. For all patients, visual evoked potential (VEP) and somatosensory evoked potential (SSEP) recordings were performed in a random order during a single session before and immediately after real or sham tDCS. All recordings were performed in the afternoon (between 14:00 and 18:00) by the same investigators (F.C. and I.B.); these investigators were not involved in recruitment, inclusion, or randomization of subjects, and had no interactions with participants prior to the examination. All recordings were numbered anonymously and analysed offline in a blinded fashion by a single investigator (G.C.), who was not blinded to the order of the blocks.

### tDCS

tDCS (2 mA, 20 min) was delivered using a constant current electrical stimulator (Brainstim®, EMSmedical) through a pair of surface electrodes: the anode was placed over the left temporal pole and the cathode was placed above the right shoulder. The electrodes were square in shape (25 cm^2^), 6-mm thick, and covered in a saline-soaked sponge. Current was delivered at a density of 0.08 mA/cm^2^, resulting in a total charge of 96 mC/cm^2^. These parameters are below the threshold for possible tissue damage [14]. During stimulation, tDCS is not usually perceived except for occasional short-lasting itching sensations below the electrodes.

The stimulation site over the left temporal pole was determined by moving laterally 40% of the intra-auricular distance from the vertex and anteriorly 5% of the distance from inion to nasion [23, 24]. The target site was located approximately halfway between the T7 and FT7 EEG positions of the international 10–20 system. This positioning method, although less accurate than neuronavigation-based techniques, adequately correlates with MRI-guided stereotactic approaches [25, 26].

For sham tDCS, the electrode positions and stimulation intensity were the same as that used for anodal stimulation, but current was only applied for the first and last 30 s of the 20-min period. This was done so that patients would not easily be able to distinguish between real tDCS and sham tDCS sessions. Participants in the sham and real arms guessed the type of stimulation in 5 and 6 cases out of 18 respectively (chi^2^ = 0.717, *p* = 1.0). The experimenters who applied tDCS (F.C. and I.B.) were also blind to the nature of the procedure (real versus sham tDCS); rather, a third experimenter (C.D.L.) pre-programmed the stimulator and ensured the randomization order.

### VEP study

Subjects were seated in a semi-dark, acoustically isolated room in front of a TV monitor surrounded by a uniform luminance field of 5 cd/m^2^. VEPs were elicited by monocular stimulation of the right eye. Visual stimuli were full-field checkerboard patterns (contrast, 80%; mean luminance, 50 cd/m^2^) generated on the TV monitor and reversed in contrast at a reversal rate of 3.1 reversals per second. The viewing distance was 114 cm and single check edges subtended a visual angle of 15 min. Subjects were instructed to fixate with their right eye on a red dot in the middle of the screen while the contralateral eye was covered with a patch. VEPs were recorded from the scalp through silver cup electrodes positioned at Oz (active electrode) and at Fz (reference electrode as per the international 10–20 system). A ground electrode was placed on the right forearm. Signals were amplified by Digitimer™ D360 pre-amplifiers (band-pass, 0.05–2000 Hz; gain, 1000) and recorded on a CED™ power 1401 device (Cambridge Electronic Design Ltd., Cambridge, UK). A total of 600 consecutive sweeps (sweep duration, 200 ms) were collected and sampled at 4000 Hz. After offline application of a 100-Hz low-pass digital filter, cortical responses were partitioned into 6 sequential blocks of 100 (including at least 95 artefact-free sweeps). Responses in each block were averaged offline (block averages) using the Signal™ software package version 3.10 (CED Ltd). VEP latencies (N1, P1, and N2) and amplitudes (N1-P1 and P1-N2) were identified. Habituation was defined as the slope of the linear regression line for the 6 blocks.

### SSEP study

SSEPs were elicited by electrical stimulation of the right median nerve at the wrist using a constant current square wave pulse (width, 0.1 ms; cathode proximal) with a stimulus intensity of 1.5-times the motor threshold and a repetition rate of 4.4 Hz. The active electrodes were placed over the contralateral parietal area (C3’, 2 cm posterior to C3 as per the international 10–20 system; referenced to Fz), over the fifth cervical spinous process (Cv5; referenced to Fz), and over Erb’s point ipsilateral to the stimulus (referenced to the contralateral side). The ground electrode was placed on the right arm. SSEP signals were amplified and recorded with the same hardware/software equipment described above for VEP recording.

Subjects were seated in a comfortable chair in a well-lit room with their eyes open. Subjects were asked to fix their attention on the stimulus-induced thumb movement. During continuous median-nerve stimulation at the wrist, 500 sweeps (sweep duration, 50 ms) were collected and sampled at 5000 Hz. A total of 500 artefact-free evoked responses were recorded and averaged for each subject (grand average). After digital filtering of the signal between 0 and 450 Hz, various SSEP components (N9, N13, N20, P25, and N33) and their respective peak-to-peak amplitudes (N9-p, N13-p, N20-P25, and P25-N33) were identified. Thereafter, based on the observation of a habituation effect from the 2nd block of 100 averaged responses onwards in previous studies [27, 28], the first 200 evoked responses were partitioned into 2 sequential blocks of 100 (including at least 95 artefact-free sweeps). Each block was averaged offline (block averages) and analysed for N20–P25 amplitudes. Habituation was expressed as the slope of the linear regression line for the 2 blocks [28].

For both VEPs and SSEPs, artefacts were automatically rejected using the Signal™ artefact rejection tool if the signal amplitude exceeded 90% of the analogue-to-digital converter (ADC) range. Signal was corrected offline for DC drift.

### Statistical analysis

Data were collected and analysed in a blinded fashion by a single investigator (V.P.) using Statistica for Windows (StatSoft Inc., Tulsa, USA) version 8.0 software. Sample size calculations were based on a ketogenic diet clinical trial that examined the same evoked potentials [29] with a desired power of 0.80 and an α error of 0.05. Since our primary endpoint was to discover differences between the effects of real and sham tDCS on habituation, we used the amplitude habituations of the N1–P1 VEP and N20–P25 SSEP cortical components in the 2 conditions (before versus after ketogenic diet) to compute the sample size. The minimal required sample size was calculated to be 16 subjects for VEP habituation and 9 subjects for SSEP habituation.

A Kolmogorov-Smirnov test showed that VEP and SSEP component latencies and amplitudes had a normal distribution. General linear models approach was used to analyse the ‘between-factor’ × ‘within-factors’ interaction effect. The between-subject factor was ‘group’ (real tDCS versus sham tDCS) or ‘time’ (before stimulation versus after stimulation) and the within-subject factor was ‘block’. Three models of repeated measures analysis of variance (ANOVA), two for VEPs (N1-P1 and P1-N2) and another for SSEPs, followed by univariate ANOVA, were used to investigate the interaction effect. Moreover, in order to analyse the slope of the linear regression (as a measure of habituation), we used a rm.-ANOVA with the between-subject factor ‘group’ (real tDCS versus sham tDCS) and the within-subject factor ‘time’ (before stimulation versus after stimulation). Univariate results were analysed only if Wilk’s Lambda multivariate significance criterion was achieved. The sphericity of the covariance matrix was verified with the Mauchly Sphericity Test; in the case of violation of the sphericity assumption, the Greenhouse-Geisser epsilon adjustment was used.

In the rm.-ANOVA and ANOVA models, partial eta2 (η_p^2^) and observed power (op) were used as measures of effect size and power, respectively. To identify the comparison(s) contributing to major effects, we performed post hoc Tukey Honest Significant Difference (HSD) tests.

One-way ANOVA tests were used to compare the baseline grand averaged VEP and SSEP latencies and amplitudes between sham and real tDCS. Paired-sample t tests were used to compare the grand averaged VEP and SSEP latencies and amplitudes before vs. after both sham and real tDCS. *P* values less than 0.05 were considered statistically significant.

## Results

### Basic neurophysiological parameters

VEP and SSEP recordings were obtained from all participants. The grand averaged VEP latencies (N1, P1, and N2; Table 2) and SEP latencies (N9, N13, N20, P25, and N33; Table 3) as well as their corresponding amplitudes (VEP: N1–P1 and P1–N2; SSEP: N9, N13, N20–P25, and P25–N33) were not significantly different between real and sham tDCS groups (*P* > 0.05). Before stimulation, both groups showed positive slope values indicating a lack of habituation in response to visual (N1–P1: real tDCS = +0.112, sham tDCS = +0.059; P1–N2: real tDCS = +0.055, sham tDCS = +0.039) and somatosensory (real tDCS = +0.448, sham tDCS = +0.234) repetitive stimulations.

**Table 2:** Latencies (in milliseconds) and amplitudes (μV) of VEPs in migraine patients’ groups undergoing real or sham transcranial direct current stimulation (tDCS) before and after intervention

*Electrophysiological parameters*	Real (*n* = 18)		Sham (*n* = 18)	
	Before	After	Before	After
N1	80.3 ± 5.7	78.9 ± 6.4	78.4 ± 2.0	78.5 ± 3.1
P1	105.5 ± 6.1	105.2 ± 5.8	105.1 ± 4.3	106.7 ± 4.7
N2	146.1 ± 8.9	146.9 ± 9.7	150.7 ± 6.7	151.1 ± 6.8
N1-P1 1st amplitude block (μV)	8.3 ± 3.1	8.9 ± 3.6	7.2 ± 2.7	6.7 ± 2.4
N1-P1 amplitude slope	0.112 ± 0.315	- 0.236 ± 0.339 **	0.059 ± 0.241	0.038 ± 0.182
P1-N2 1st amplitude block (μV)	8.3 ± 3.1	8.9 ± 4.2	6.4 ± 3.4	6.3 ± 2.9
P1-N2 amplitude slope	0.055 ± 0.507	- 0.345 ± 0.569	0.039 ± 0.272	- 0.001 ± 0.269

Data are expressed as means ± SD. ** = *p* < 0.01 before vs. after the intervention

**Table 3:** Grand-average somatosensory evoked potentials (SSEPs) latencies and amplitudes in migraine patients’ groups undergoing real or sham transcranial direct current stimulation (tDCS) before and after intervention

*Electrophysiological parameters*	Real (*n* = 18)		Sham (*n* = 18)	
	Before	After	Before	After
N9 (ms)	9.5 ± 0.6	9.7 ± 0.8	9.5 ± 0.6	9.6 ± 0.6
N13 (ms)	13.2 ± 0.8	13.3 ± 0.8	13.1 ± 0.7	13.2 ± 0.7
N20 (ms)	18.8 ± 0.9	19.0 ± 0.8	18.6 ± 1.1	18.8 ± 1.1
P25 (ms)	23.6 ± 2.2	23.9 ± 2.1	22.9 ± 2.2	23.2 ± 2.2
N33 (ms)	31.5 ± 2.6	31.5 ± 1.6	31.9 ± 2.1	31.5 ± 1.3
N9-p (μV)	4.1 ± 1.6	3.8 ± 1.4	3.5 ± 1.4	3.5 ± 1.9
N13-p (μV)	2.0 ± 0.8	2.0 ± 0.6	2.0 ± 0.6	1.8 ± 0.7
N20-P25 (μV)	2.3 ± 1.3	2.4 ± 1.5	2.3 ± 0.7	2.1 ± 0.9
P25-N33 (μV)	1.3 ± 0.5	1.3 ± 0.9	1.2 ± 0.5	1.0 ± 0.5
N20-P25 1st amplitude (μV)	2.4 ± 1.1	2.2 ± 1.2	2.3 ± 0.7	2.2 ± 0.6
N20-P25 amplitude slope	0.448 ± 0.710	0.315 ± 0.543	0.234 ± 0.406	0.213 ± 0.481

Data are expressed as means ± SD

### Effects of tDCS on neurophysiological parameters

The grand averaged VEP latencies (N1, P1, and N2; Table 2) and SSEP latencies (N9, N13, N20, P25, and N33; Table 3) as well as their corresponding amplitudes (VEP: N1–P1 and P1–N2; SSEP: N9, N13, N20–P25, and P25–N33) were not significantly different before and after stimulation in both the real and sham tDCS groups (*P* > 0.05).

**Fig. 1:**
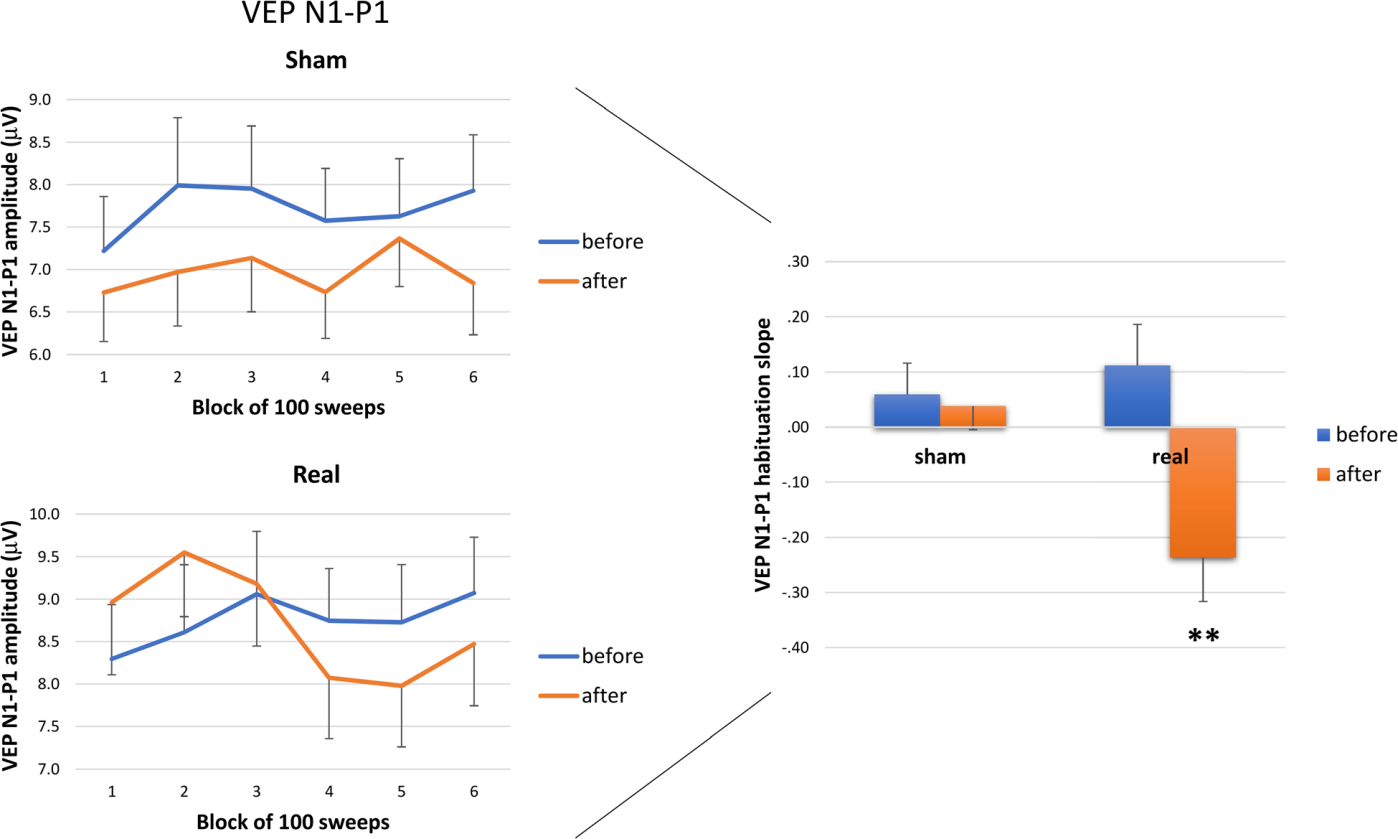
Left panel: Amplitudes (mean ± standard error of the mean) of the N1–P1 visual evoked potential (VEP) component in 6 sequential blocks of 100 recordings are shown before and after sham tDCS (upper panel) and anodal tDCS (lower panel). Right panel: The bar graph represents the habituation slope of VEP N1–P1 peak-to-peak amplitudes (mean ± standard error of the mean) before and after sham tDCS and real tDCS. The arrow highlights interictal VEP habituation that was reduced before real tDCS but normalized after. ** = *p* < 0.01 before vs. after the intervention

In the rm.-ANOVA model using the VEP N1–P1 peak-to-peak block amplitude as the dependent variable, the multivariate test was significant for the ‘group’ × ‘time’ × ‘block’ interaction effect (F_5,340_ = 3.290, *p* = 0.006). The univariate rm.-ANOVA for N1–P1 peak-to-peak amplitudes confirmed a significant interaction factor effect (Greenhouse-Geisser epsilon adjustment applied, F_4.1282.1_ = 3.29, ε = 0.83, *p* = 0.01, partial η2 = 0.05, op = 0.89) in the multivariate test. At the post-hoc analysis 1st N1-P1 VEP amplitude block did not differ between before and after both stimulations. The linear regression N1–P1 slope of VEP amplitudes over all blocks was significantly different between before and after stimulation (F_1,34_ = 5.21, *p* = 0.029, partial η2 = 0.133, op = 0.60; raw data are shown in Fig. 1). A post-hoc analysis showed that the slope of VEP amplitudes from block 1 to block 6 was positive before the intervention in both the real tDCS (+0.112) and sham tDCS (+0.059) groups, whereas after the intervention these values were negative in the real tDCS group (−0.236, *p* = 0.003 versus before stimulation) but positive in the sham tDCS group (+0.038, *p* > 0.05 versus before stimulation) (Fig. 1, right panel).

**Fig. 2:**
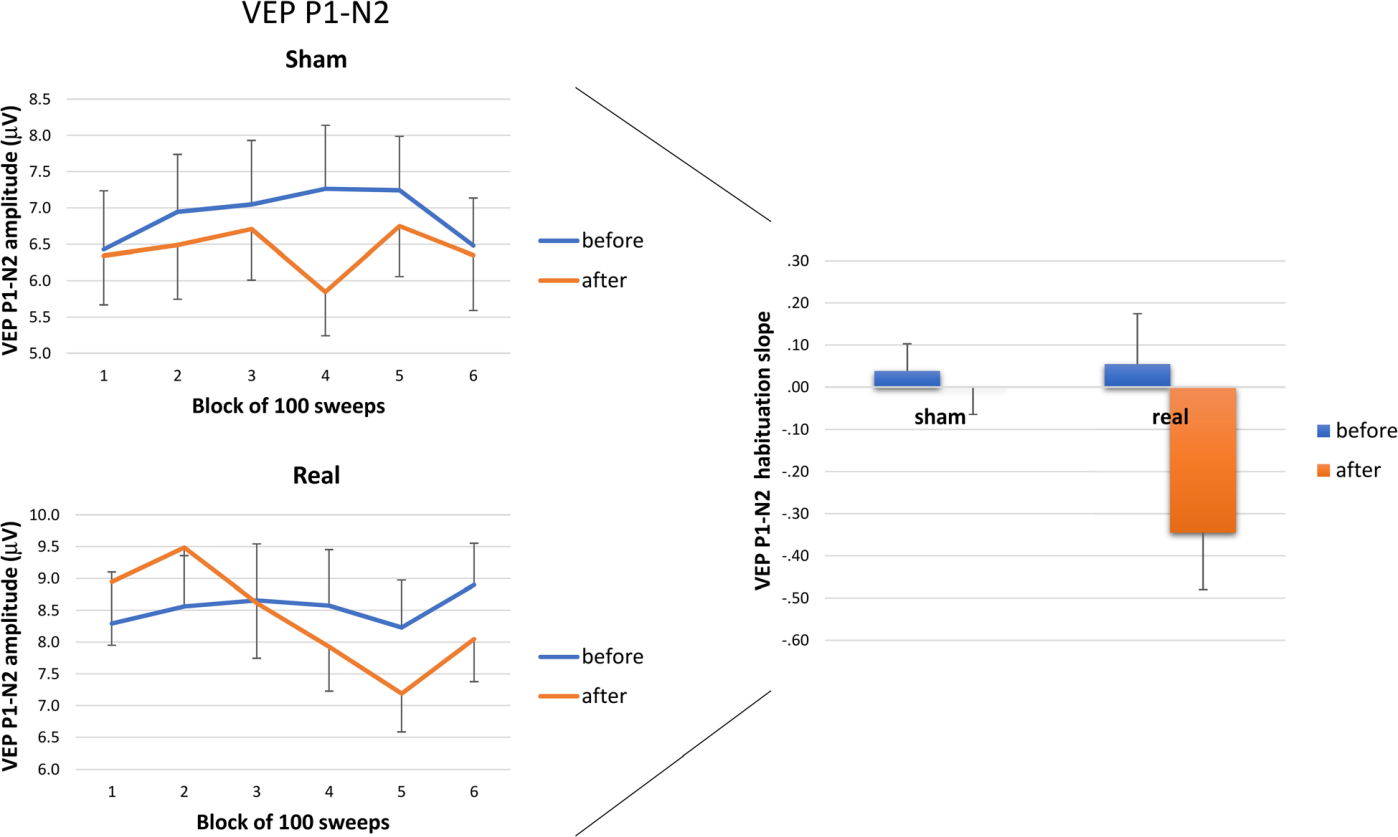
Left panel: Amplitudes (mean ± standard error of the mean) of the P1–N2 visual evoked potential (VEP) component in 6 sequential blocks of 100 recordings are shown before and after sham tDCS (upper panel) and real tDCS (lower panel). Right panel: The bar graph represents the habituation slope of VEP P1–N2 peak-to-peak amplitudes (mean ± standard error of the mean) before and after sham tDCS and real tDCS

In the rm.-ANOVA model using the VEP P1–N2 peak-to-peak block amplitude as the dependent variable, the ‘group’ × ‘time’ × ‘block’ interaction effect was not significant (F_5,340_ = 1.55, *p* = 0.171) in the multivariate test (Fig. 2).

**Fig. 3:**
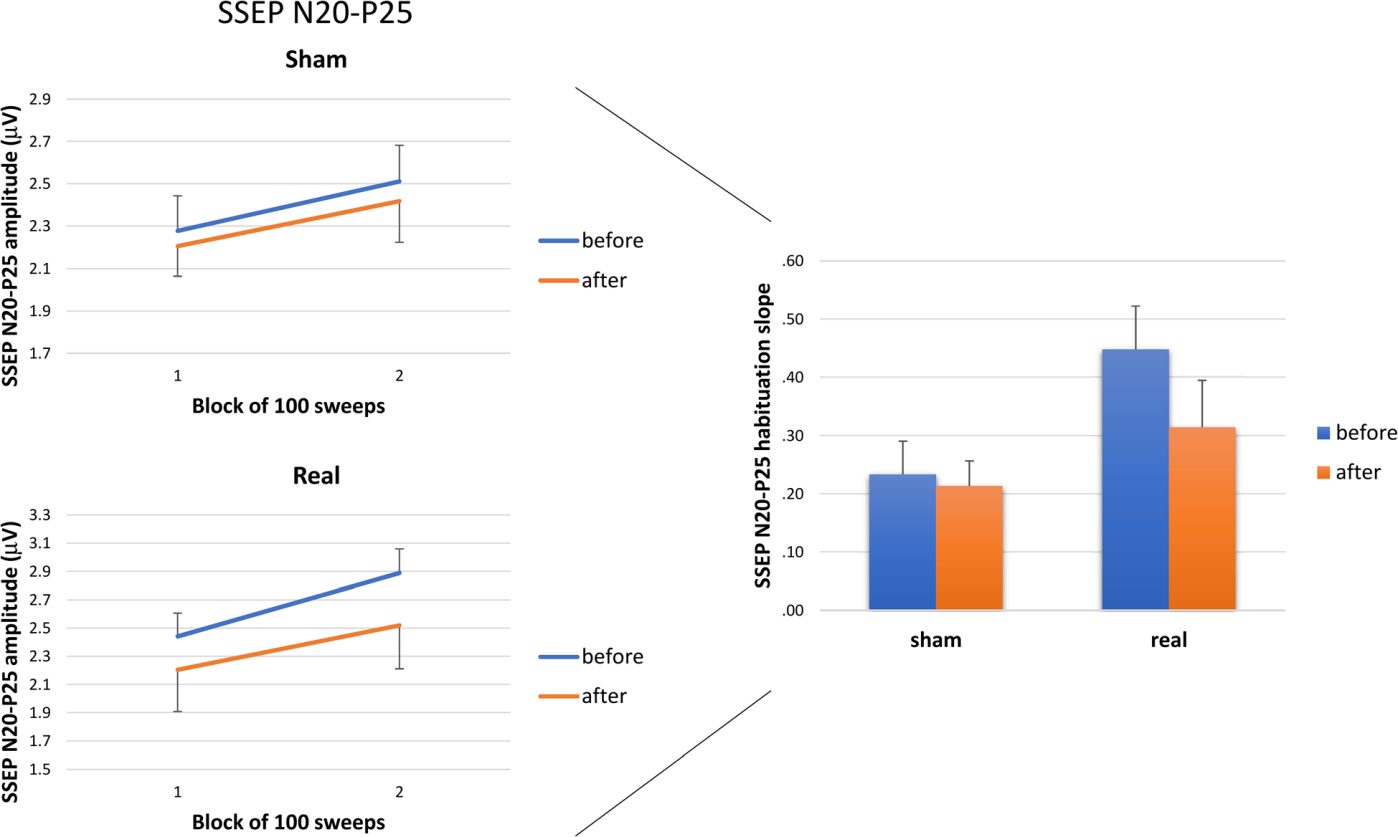
Left panel: Amplitudes (mean ± standard error of the mean) of the N20–P25 somatosensory evoked potential (SSEP) component in 2 sequential blocks of 100 recordings are shown before and after sham tDCS (upper panel) and real tDCS (lower panel). Right panel: The bar graph represents the habituation slope of SSEP N20–P25 peak-to-peak amplitudes (mean ± standard error of the mean) before and after sham tDCS and real tDCS

In the rm.-ANOVA model using the SSEP N20–P25 peak-to-peak block amplitude as the dependent variable, the ‘group’ × ‘time’ × ‘block’ interaction effect was not significant (F_1,68_ = 0.19, *p* = 0.659) in the multivariate test (Fig. 3).

## Discussion

The present study mainly revealed that a single session of anodal tDCS over the left temporal pole restored normal visual but not somatosensory habituation in interictal migraineurs.

Neurophysiological studies have shown that interictal migraineurs exhibit dysfunctional sensory information processing in the form of habituation deficits in response to various sensory inputs, including visual and somatosensory inputs [2]. Recent neuroimaging studies have revealed subtle microstructural alterations in the brains of patients with migraine in areas associated with the ictal-interictal cycle. Among these studies, some evidence highlights a pathophysiological role for the TP in migraine [7–12].

The TP region encompasses the most anterior segment of the temporal lobe and receives extensive inputs from visual regions of the thalamus [30, 31]. Additionally, the TP is highly interconnected with the amygdala, hippocampus, superior temporal gyrus, hypothalamus, occipitobasal cortex, prefrontal regions, and insula, suggesting its participation in autonomic regulation, memory, and emotional processing [32, 33]. The TP is considered a multisensory associative cortex because it is also connected to the main sensory systems of the temporal lobe, including the visual, auditory, olfactory, and gustative systems, but not the somatosensory system [32, 34]. Indeed, neuroimaging studies have demonstrated subregional activation of the TP in response to specific sensory stimuli, with the ventromedial aspect of the TP having a predominant role in higher order visual information processing [34] as part of the VVS.

Our finding that anodal (excitatory) stimulation of the left TP restored physiological visual information processing but not somatosensory processing in interictal migraineurs is largely consistent with the abovementioned roles of the left TP in high-level multimodal perceptual processing. A selective effect of tDCS over the TP on visual information processing is probably related to the role of the TP in the VVS and its lack of participation in somatosensory elaboration. Interestingly, another study observed similar normalization of abnormal interictal VEP habituation in response to the application of tDCS over the occipital cortex in migraineurs [22]. This can be explained either by a direct interconnection between the TP and occipital cortex along the VVS or an indirect effect of the tDCS on brain structures that positively modulate both cortices.

The VVS is involved in visual recognition and in the assignment or retrieval of a given meaning for visual information [35]. After early activation of the occipital area, the complexity of representation of visual information increases as information flows to the anterior regions of the VVS, with the TP located at the end of the stream and sending backward facilitatory projections to the occipital cortex to optimize sensory processing (e.g., improve perception and learning) [35, 36]. Consistent with this evidence, we observed that the enhancement of TP activity with anodal tDCS improved VEP amplitude habituation, a basic form of learning [37], without affecting initial baseline excitability (reflected by non-significant changes in 1st block VEP amplitudes). In habituation paradigms, early and late responses can behave differently as a result of regulation by different mechanisms; according to the dual-process theory, increasing responsiveness (sensitization) competes with decreasing responsiveness (habituation) to determine final behavioural outcomes. Facilitation occurs at the beginning of the stimulus session and accounts for an initial temporary increase in response amplitude, whereas habituation occurs throughout the recording session and accounts for delayed decreases in responsiveness [38]. Therefore, our results regarding the selective effect of anodal tDCS on delayed habituation in migraineurs appear to be in line with the putative mechanism of tDCS; that is, the ability of tDCS to affect the potentiation of long-term learning processes and synaptic plasticity underlying learning and memory [39]. Alternatively, it has been shown that anodal tDCS exerts modulatory effects on thalamo-cortical circuits by increasing functional coupling between the thalamus and cortex [17, 40]. These experimental observations are of particular interest in migraine because independent research groups have previously reported reduced functional [41, 42] and morphological [43, 44] thalamic integrity coupled with decreased intracortical inhibition during visual stimulation in migraineurs [45, 46]. We thus can hypothesize that an alternative mechanism of action for anodal tDCS in the present study is increased thalamo-cortical activity, which in turn increased delayed inhibitory mechanisms to restore normal VEP habituation.

Irrespective of the mechanism, the observation that tDCS over the left TP is able to restore normal VEP habituation in interictal migraineurs leads to hypothesize that together with the visual, motor, and dorsolateral prefrontal cortices [19, 20], the TP could represent a novel target for tDCS as a prophylactic strategy for treating migraine [47].

This study had some limitations. For example, we only stimulated the left TP, such that we cannot know whether anodal tDCS of the right TP would have yielded similar results. Several studies have shown divergent functional roles of the left and right TP, where the right TP is more involved in elaborating socio-emotional implications of multisensory perceptual stimuli [48] while the left TP is mostly implicated in perceptual decoding, semantic processing, and conceptualization [34]. Nonetheless, both the left and right TPs are joined via the interior white commissure to advance multimodal perceptual analysis [32], such that the relevance of the right TP cannot be discounted. Furthermore, the positioning method we used is accurate, although not as accurate as neuronavigation-based techniques, which are unfortunately only available for neurosurgical procedures in our clinic. Another shortcoming of the present study is the lack of inclusion of a healthy control group undergoing the same stimulations, although this would not add anything to the results of the study because the healthy subjects usually already habituate normally at the baseline, i.e. we cannot normalize the already normal information processing.

## Conclusions

In conclusion, anodal but not sham tDCS selectively enhanced visual but not somatosensory habituation in interictal migraineurs probably by restoring normal inhibitory activity of the left TP. We propose that this effect can be explained by either a direct interference with short- and long-term synaptic plasticity mechanisms or an indirect potentiation of the thalamo-cortical circuit. Further studies are needed to determine whether TP stimulation also normalizes the habituation response to other sensory inputs, such as auditory and nociceptive inputs. Regardless of the underlying cellular and molecular mechanisms of our observed effect, we propose that the TP should be considered as a key site of involvement in the pathophysiology of migraine and as a potential therapeutic target. Clinical studies are needed to clarify whether repeated sessions of anodal tDCS improve TP function and connectivity in patients with migraine to ultimately reduce the number and severity of migraine attacks.

## Abbreviations

EEG: Electroencephalogram
MO: Migraine without aura
SSEP: Somatosensory evoked potential
tDCS: transcranial direct current stimulation
TP: Temporal pole
VEP: Visual evoked potential
VVS: Ventral visual stream
